# Editorial: Rising stars in neonatology: 2023

**DOI:** 10.3389/fped.2024.1440664

**Published:** 2024-06-28

**Authors:** Lukas P. Mileder, Janneke Dekker, Kazumichi Fujioka

**Affiliations:** ^1^ Division of Neonatology, Department of Pediatrics and Adolescent Medicine, Medical University of Graz, Graz, Austria; ^2^ Department of Pediatrics, Leiden University Medical Center, Leiden, Netherlands; ^3^ Department of Pediatrics, Kobe University, Kobe, Japan

**Keywords:** neonatology, research, scientific improvement, clinical outcome, evidence-based practice

**Editorial on the Research Topic** Rising stars in neonatology: 2023

It is acknowledged that medicine as it is being practiced today is an applied science. This is substantiated by all the scientific progress over the last decades and the subsequent progress in clinical practice (1). Nonetheless, this does not derogate the need for further diligently planned and executed empirical studies—studies that are relevant, cost-efficient, and ethically sound—as these have the potential not only to inform and impact clinical practice but also to improve outcomes for newborn infants and their families (2). Scientific evidence forms the basis of recommendations for essential interventions in neonatology, for example, neonatal resuscitation after birth (3) or the management of respiratory distress syndrome (4). However, to provide sound treatment recommendations, we need scientific evidence that has focused on appropriate (clinical) outcomes. The development of a core outcome set for neonatal research should further support scientists' outcome selection, ensuring that studies are targeting the issues most relevant to providers and their patients (5).

Driven by advances in several scientific fields including genetics and advanced imaging, there have been increasing opportunities for research in neonatal-perinatal medicine (6). Consequently, clinical studies in the field of neonatology are as multifaceted as ever (7). This scientific plurality is also represented by the articles in this Research Topic, which is aimed at showcasing the high-quality work of internationally recognized researchers in the early stages of their careers. The first two studies in this Research Topic have used scientific advantages, one in genetics and advanced laboratory analytics and the other in non-invasive patient monitoring, for their respective investigations:

Vidinopoulos et al. (Front Pediatr 2023) studied the effects of 24 h of mechanical ventilation on inflammation in the brainstem respiratory centers of preterm fetal sheep. They found increased systemic interleukin levels in comparison to the control group of non-ventilated sheep but only moderate inflammatory effects—and similar mRNA expression levels of inflammation, injury, cell death, and prostaglandin synthesis—within the brainstem respiratory centers of the ventilated sheep.

In their *post hoc* analysis of data from previous observational studies, Wolfsberger et al. (Front Pediatr 2023) targeted fractional tissue oxygen extraction, i.e., relative oxygen extraction from the arterial to the venous compartment, in peripheral muscle tissue, as calculated from the tissue oxygenation index measured by near-infrared spectroscopy. Based on data from 240 stable term and late preterm neonates, they were able to provide reference values of peripheral fractional tissue oxygen extraction for the first 24 h after birth, which remained stable when comparing four 6-h periods.

Evaluating the quality of medical care is often the first step to identifying areas for improvement. Wudu et al. (Front Pediatr 2024) prospectively studied 306 neonates admitted to neonatal intensive care units in Northeast Ethiopia due to sepsis, focusing on mortality and predictors of time to death. While the mortality rate was alarmingly high, the identified modulating factors such as tachypnea, thrombocytopenia, and use of continuous positive airway pressure could serve as catalysts for improving care practices and patient survival.

Finally, as part of our family-centered approach, especially in neonatology and all of pediatrics, we must as clinical researchers always bear in mind how important it is to actively work together with parents while planning and doing research (8). Tippmann et al. (Front Pediatr 2024) investigated parental perceptions of informed consent procedures for neonatal research, with their results emphasizing the importance of gaining parental approval of study participation, but also showing that the timing of consent obtainment—before or after birth—was of less importance to parents.

While pre-clinical and clinical studies are important first steps, improving the quality of neonatal care requires active dissemination, implementation in guidelines and policies, and, ultimately, adoption in practice (9). Therefore, while we hope that this research collection is informative and worth reading, we hope even more that these studies will help inform further research as well as clinical practice to come one step closer to our joint goal of improving the health and quality of life of our patients.
